# Co‐targeting FAK and Gli1 inhibits the tumor‐associated macrophages‐released CCL22‐mediated esophageal squamous cell carcinoma malignancy

**DOI:** 10.1002/mco2.381

**Published:** 2023-10-15

**Authors:** Jie Chen, Yanmeng Zhu, Di Zhao, Lingyuan Zhang, Jing Zhang, Yuanfan Xiao, Qingnan Wu, Yan Wang, Qimin Zhan

**Affiliations:** ^1^ Key Laboratory of Carcinogenesis and Translational Research (Ministry of Education/Beijing) Laboratory of Molecular Oncology Peking University Cancer Hospital & Institute Beijing China; ^2^ Peking University International Cancer Institute Peking University Beijing China; ^3^ Research Unit of Molecular Cancer Research Chinese Academy of Medical Sciences Beijing China; ^4^ Soochow University Cancer Institute Suzhou China; ^5^ Institute of Cancer Research Shenzhen Bay Laboratory Shenzhen China

**Keywords:** CCL22, esophageal squamous cell carcinoma, FAK, Gli1, malignancy, tumor‐associated macrophages

## Abstract

Esophageal squamous cell carcinoma (ESCC) is a frequently seen esophageal tumor type in China. Activation of signaling proteins and relevant molecular mechanisms in ESCC are partially explored, impairing the antitumor efficiency of targeted therapy in ESCC treatment. Tumor‐associated macrophages (TAMs)‐released C‐C motif chemokine 22 (CCL22) can activate intratumoral focal adhesion kinase (FAK), thus promoting the progression of ESCC. Here, we demonstrated that highly secreted CCL22 by TAMs (CCL22‐positive TAMs) induced ESCC cell stemness and invasion through facilitating transcriptional activity of intratumoral glioma‐associated oncogene 1 (Gli1), a downstream effector for Hedgehog (HH) pathway. Mechanistically, FAK‐activated protein kinase B (AKT) mediated Gli1 phosphorylation at its Ser^112/^Thr^115/^Ser^116^ sites and released Gli1 from suppressor of fused homolog, the endogenous inhibitor of Gli1 to activate downstream stemness‐associated factors, such as SRY‐box transcription factor 2 (SOX2), Nanog homeobox (Nanog), or POU class 5 homeobox (OCT4). Furthermore, inhibition of FAK activity by VS‐4718, the FAK inhibitor, enhanced antitumor effect of GDC‐0449, the HH inhibitor, both in xenografted models and in vitro assays. Clinically, CCL22/Gli1 axis is used to evaluate ESCC prognosis. Overall, our study establishes the communication of FAK with HH pathway and offers the novel mechanism related to Gli1 activation independent of Smoothened as well as the rationale for the anti‐ESCC combination treatment.

## Graphical abstract

TAMs‐released CCL22 activates intratumoral CCR4/FAK/AKT axis, which induces the phosphorylation of Gli1 Ser^112/^Thr^115/^Ser^116^ sites, and stimulates Gli1 activity to induce the stemness and metastasis of tumor cells. FAK inhibitor‐VS‐4718 enhanced the antitumor effect of HH inhibitor‐GDC‐0449. Our study offers the novel mechanism related to Gli1 activation independent of SMO as well as the rationale for the anti‐ESCC combination treatment.

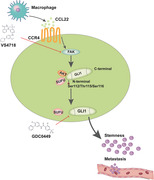

## 1 INTRODUCTION

Esophageal cancer (EC) has posed a tremendous threat to human health. Esophageal squamous cell carcinoma (ESCC) occupies 90% of global EC cases, which shows a high prevalence rate in China.^1^, ^2^ Extensive treatments are needed for many ESCC cases, like surgery, chemotherapy, and chemoradiotherapy. Nowadays, targeted and immuno‐oncology treatments exhibit potential early effects on ESCC.^3^, ^4^, ^5^, ^6^ Some large sequencing and multi‐platform studies assess the epigenetic, transcriptomic, and mutational patterns of ESCC. Several dysregulated molecules, such as receptor tyrosine‐protein kinase erbB‐2 (ERBB2), epidermal growth factor receptor (EGFR), vascular endothelial growth factor A (VEGFA), insulin‐like growth factor 1 receptor (IGF1R), phosphatidylinositol 4,5‐bisphosphate 3‐kinase catalytic subunit alpha isoform (PIK3CA), and fibroblast growth factor receptor 1 (FGFR1), have been identified.^7^, ^8^, ^9^, ^10^

Activation of intratumoral signaling molecules by tumor microenvironment (TME) is the major mechanism that contributes to ESCC malignancy.^11^, ^12^, ^13^ Our previous work has shown that tumor‐associated macrophages (TAMs)‐released C‐C motif chemokine 22 (CCL22) stimulates the activity of its receptor‐C‐C chemokine receptor type 4 (CCR4) in ESCC cells and subsequently activates the intratumoral focal adhesion kinase (FAK)/AKT axis to induce the malignant progression of ESCC.^14^ FAK plays a role in signal translation from extracellular matrix components and/or TME to intracellular tumor‐promoting pathways, and FAK may have a critical role in mediating TME‐induced activation of intratumoral signaling pathways.^15^, ^16^, ^17^, ^18^ Importantly, FAK mediates complex effects of the tumor immune microenvironment and tumor cells via activating transcriptional factors and then inducing the expression or secretion of downstream tumor‐promoting molecules and TME‐educating factors. Collectively, choosing FAK as the effective target is critical for the combination therapy, especially if tumor cells rely on the intercellular signalings in the milieu of TME. Multiple signaling pathways are usually manipulated by the interaction between tumor cells and their surrounding TME to control several steps during tumor malignant progression, but how they cooperate with one another to control relevant biologic behaviors and whether these signaling interactions can be targetable remain poorly understood.

The hedgehog (HH) signaling is overactivated in tumor cells and correlated with tumorigenesis.^19^, ^20^, ^21^ Secreted HH mediates G‐coupled receptor‐like signal transducer Smoothened (SMO) to activate Gli, followed by nuclear translocation and stimulation of tumor‐promoting gene transcription.^22^ Interestingly, glioma‐associated oncogene 1 (Gli1) can be activated by phosphatidylinositol 3 kinase (PI3K)/AKT pathway in mesenchymal stromal cells,^23^ critical for immune response in macrophages regulated by Foxo1/β‐catenin axis,^24^ and stimulated by mammalian target of rapamycin (mTOR)/ribosomal protein S6 kinase 1 (S6K1) signaling cascade in chondrosarcoma.^25^ These studies have shown that Gli1 can be activated by several intracellular signaling proteins independent of the canonical HH signaling, indicating that Gli1 may possibly be the excellent candidate for combination therapy. Importantly, Gli1 was tightly correlated with the malignancy of ESCC according to previous studies.^26^, ^27^, ^28^ The more complete understanding toward Gli1‐regulating mechanisms in ESCC could be beneficent for ESCC‐targeted therapies.

Although both FAK and Gli1 are the critical targets for the development of antitumor agents, the correlation between intratumoral FAK and HH/Gli1 pathways in ESCC, especially in the presence of TME, has not been yet explored. In the present study, we attempted to classify the combinatorial effect of FAK and HH/Gli1 pathways inhibition on the ESCC malignancy. Correspondingly, we explored whether the CCL22/FAK signaling axis related to HH pathway activation in ESCC and the underlying mechanisms.

## 2 RESULTS

### 2.1 CCL22‐positive TAMs facilitate ESCC malignancy via intratumoral CCR4

**FIGURE 1:**
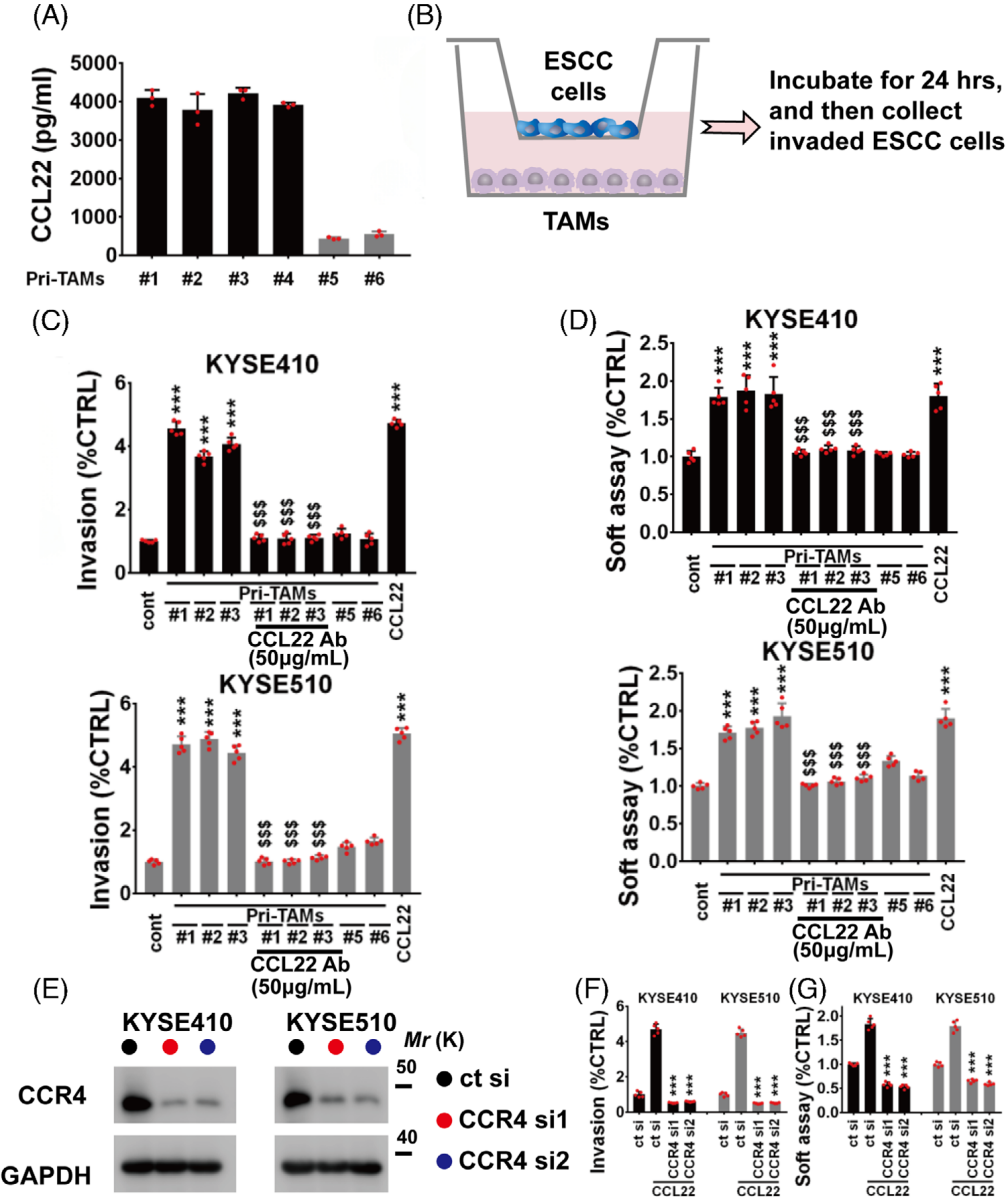
C‐C motif chemokine 22 (CCL22) **promotes** esophageal squamous cell carcinoma (ESCC) **progression**. (A) The secretion of CCL22 from primary tumor‐associated macrophages (PriTAMs) was evaluated using enzyme‐linked immunosorbent assay (ELISA). (B) Protocols of invasion assay. (C) Invasive ability of KYSE410 (upper panel) or KYSE510 (lower panel) cells in the presence of CCL22‐positive PriTAMs with/without CCL22 Ab, or CCL22‐negative PriTAMs or rhCCL22 (50 ng/mL). (D) Soft agar‐based colony formation of KYSE410 (upper panel) or KYSE510 (lower panel) cells treated with conditioned media (CM) from CCL22‐positive PriTAMs with/without CCL22 Ab, or CCL22‐negative PriTAMs, or with rhCCL22 (50 ng/mL). (E) KYSE410 and KYSE510 cells were transfected with CCR4 siRNAs, and the transfection efficiency was evaluated using immunoblotting. Glyceraldehyde‐3‐phosphate dehydrogenase (GAPDH) served as internal control. (F–G) The invasive or anchorage‐independent growth ability of indicated ESCC cells in the presence of rhCCL22 (50 ng/mL) was evaluated using transwell invasion assay (F) or soft agar‐based colony formation assay (G). ^***^ *p* < 0.001 as compared with the control cells. ^$$$^ *p* < 0.001 as compared with PriTAMs #1, #2, or #3. Two‐tailed unpaired Student's *t*‐test. Error bars, mean ± SD of five independent experiments.

Previously, we have demonstrated that TAMs‐derived CCL22 is abundantly expressed in ESCC stroma.^14^ We evaluated the secretion status of CCL22 in six cases of primary TAMs using enzyme‐linked immunosorbent assay (ELISA) assay and found that CCL22 is highly expressed in the conditioned media (CM) of four cases of primary TAMs (CCL22‐positive TAMs), whereas the secretion of CCL22 from other two cases of primary TAMs are very low (CCL22‐negative TAMs) (Figure 1A). Next, we used co‐culture transwell system (pore size: 8 μm) for evaluating TAMs‐mediated ESCC cell invasion through culturing KYSE410/KYSE510 cells within the top transwell chamber and TAMs within the bottom chamber (Figure 1B). CCL22‐positive TAMs could effectively promote KYSE410/KYSE510 cell invasion (Figure 1C and Figure S1). Nonetheless, CCL22‐negative TAMs (two cases) could not induce KYSE410/KYSE510 cell invasion (Figure 1C and Figure S1). CCL22 Ab (50 μg/mL) evidently blocked CCL22‐positive TAMs‐mediated ESCC cell invasion (Figure 1C and Figure S1).

Metastatic tumor cells experience the epithelial‐mesenchymal transition (EMT) and acquire stem cell‐like phenotype.^29^ In our prior work, TAMs‐released CCL22 effectively induced the EMT of ESCC cells.^14^ We further assessed whether TAMs‐derived CCL22 could induce the anchorage‐independent growth of ESCC cells and found that CM from CCL22‐positive TAMs significantly stimulated the soft agar‐based colony formation of KYSE410 and KYSE510 cells, whereas the CM from CCL22‐negative TAMs not stimulated the soft agar‐based colony formation of KYSE410 and KYSE510 cells (Figure 1D). Additionally, employment of CCL22 Ab (50 μg/mL) effectively suppressed CCL22‐positive TAMs‐induced anchorage‐independent colony forming ability in indicated ESCC cells (Figure 1D).

CCR4 expressed in ESCC cells has an important effect on CCL22‐induced FAK/AKT pathway activation.^14^ We depleted CCR4 in KYSE410/KYSE510 cells with siRNAs to observe whether CCR4 is critical for CCL22‐mediated ESCC malignant progression. As the results shown in Figure 1E–G and Figure S2, depletion of intratumoral CCR4 effectively blocked recombinant human (rh) CCL22 protein (50 ng/mL)‐mediated invasion and anchorage‐independent growth of KYSE410 and KYSE510 cells.

### 2.2 CCL22 induces tumor malignancy via stimulating the intratumoral Gli1 activity

**FIGURE 2:**
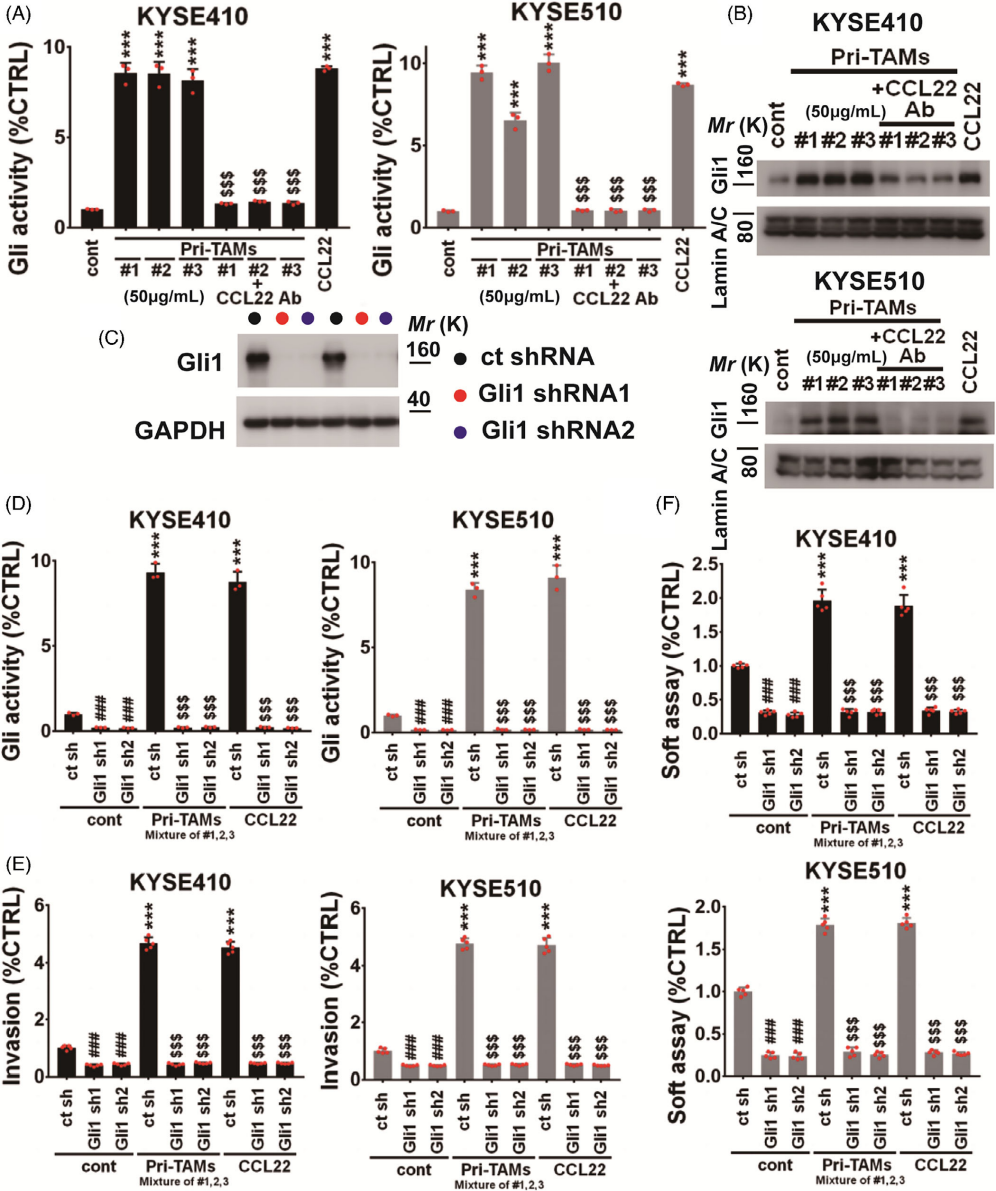
C‐C motif chemokine 22 (CCL22) **facilitates** esophageal squamous cell carcinoma (ESCC) **malignancy via activating intratumoral** glioma‐associated oncogene 1 (Gli1). (A) Luciferase reporter assay showing that the Gli1 activity in indicated KYSE410 (left panel) or KYSE510 (right panel) cells. ***p < 0.001 as compared with the control cells. ^$$$^ *p* < 0.001 as compared with primary tumor‐associated macrophages (PriTAMs) #1, #2, or #3. (B) Immunoblotting assay showing that nuclear expression of Gli1 in indicated KYSE410 (upper panel) or KYSE510 (lower panel) cells. Lamin A/C was served as internal control. (C) KYSE410 and KYSE510 cells were stably transfected with control shRNA or Gli1 shRNA1 and 2. The transfection efficacy was evaluated using immunoblotting for detection of Gli1 expression, and GAPDH was used as loading control. (D) The Gli activity of indicated KYSE410 (left panel) or KYSE510 (right panel) cells was assessed using luciferase reporter assay. (E) Invasive ability of indicated KYSE410 (left panel) or KYSE510 (right panel) cells. (F) Soft agar‐based colony formation of indicated KYSE410 (upper panel) or KYSE510 (lower panel) cells. (D–G) ***p < 0.001 as compared with the control cells. ^$$$^ *p* < 0.001 as compared with primary tumor‐associated macrophages (PriTAMs) or rhCCL22 (50 ng/mL). ^###^ *p* < 0.001 as compared with control cells. Two‐tailed unpaired Student's *t*‐test. Error bars, mean ± SD of three to five independent experiments.

Since Gli1 is the important tumor‐promoting transcriptional factor and therapeutic target, we utilized the Gli‐dependent luciferase reporter system for evaluating effects of TAMs‐released CCL22 on the intratumoral Gli1 activity and its nuclear location. CM from CCL22‐positive TAMs and rhCCL22 protein (50 ng/mL) substantially increased the Gli transcriptional activity and the nuclear Gli1 expression (Figure 2A,B). To further evaluate whether CCR4 participates in CCL22‐mediated Gli1 activity, rhCCL22 (50 ng/mL) was added to incubate with CCR4‐depleted KYSE410/KYSE510 cells, we found that CCR4 siRNAs effectively inhibited CCL22‐induced expression of nuclear Gli1 and its activity in indicated ESCC cells (Figure S3A,B).

We investigated whether Gli1 activation is required for the CCL22 signaling‐mediated invasiveness of ESCC cells, found that depletion of Gli1 by short‐hairpin (sh)RNA blocked the activity of Gli reporter induced by rhCCL22 and pri‐TAMs (Figure 2C,D), and inhibited the CCL22‐ or TAMs‐induced anchorage‐independent colony formation and invasion of ESCC cells (Figure 2E,F and Figure S4). Correspondingly, CM from CCL22‐positive TAMs and rhCCL22 (50 ng/mL) effectively upregulated stemness‐related effector levels, like SRY‐box transcription factor 2 (SOX2), Nanog homeobox (Nanog), or POU class 5 homeobox (OCT4) in KYSE410 and KYSE510 cells; however, Gli1 shRNA blocked the CCL22‐stimulated expression of these stemness‐related markers (Figure S5A–C). Therefore, Gli1 activation has functional involvement in CCL22 signaling.

We then evaluated Gli1 levels in 114 ESCC specimens through immunohistochemistry (IHC) assay. Seventy‐six out of the 114 cases (67%) for Gli1 were positive. However, 14 out of the 66 cases (21%) for Gli1 staining in adjacent normal tissues were positive (Figure S6A). There was strongly positive correlation between the level of intratumoral Gli1 and some clinical factors, like T/N status or tumor stage (Figure S6B). As revealed by results of Kaplan–Meier curve analysis, ESCC patients harbored high Gli1 level that exhibited poor overall survival (Figure S6C). We analyzed the clinical relevance between Gli1 and stromal CCL22, and found that Gli1 level was tightly related to stromal CCL22 expression in 45 cases of ESCC tissue samples (Figure S6D).

### 2.3 CCL22 promotes Gli1 activity via FAK/AKT axis

**FIGURE 3:**
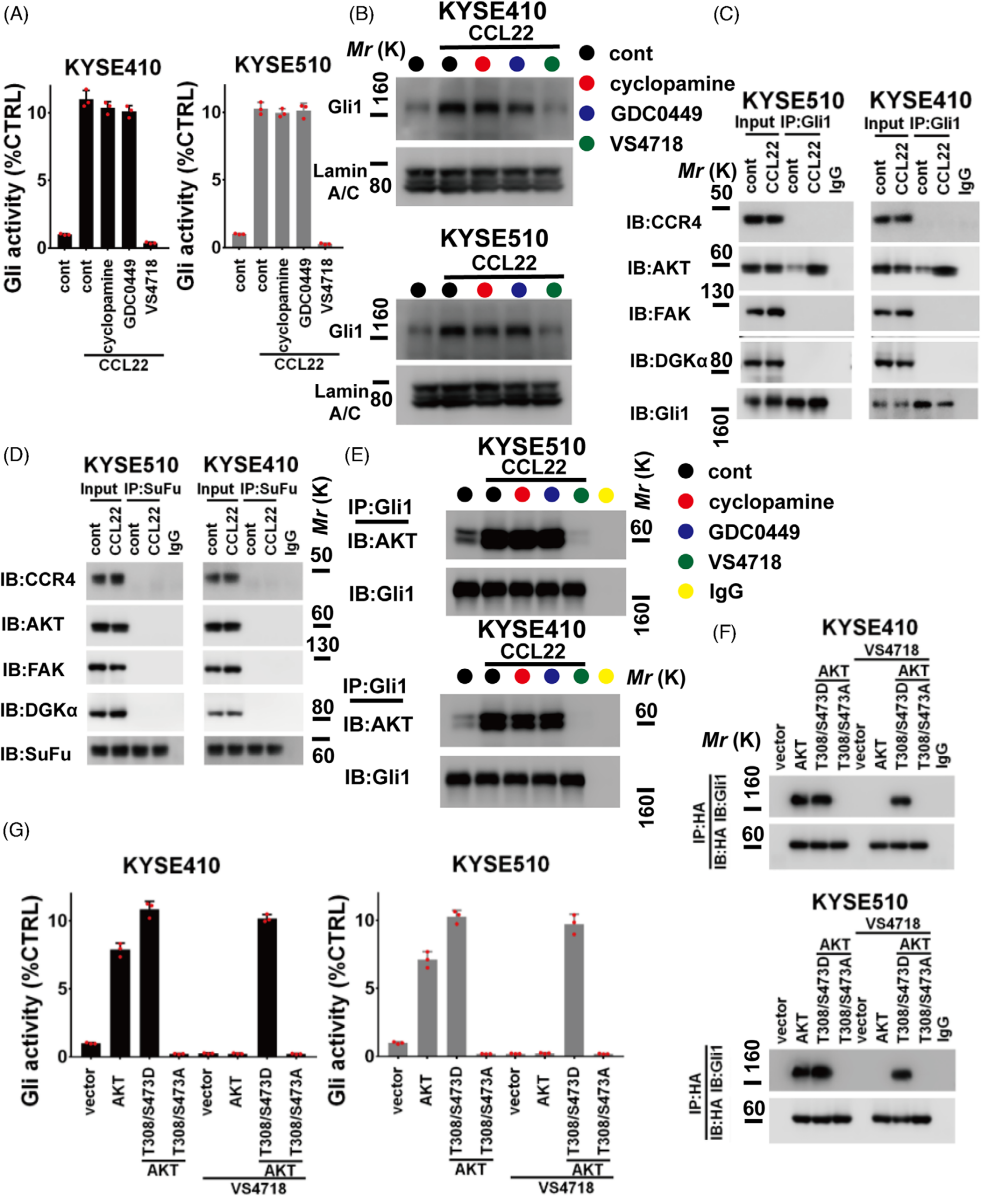
C‐C motif chemokine 22 (CCL22) **activates intratumoral** focal adhesion kinase (FAK)**/**glioma‐associated oncogene 1 (Gli1) **axis**. (A) Luciferase reporter assay for Gli transcriptional activity in indicated KYSE410 (left panel) or KYSE510 (right panel) cells. (B) Immunoblotting assay for nuclear level of Gli1 in indicated KYSE410 (upper panel) or KYSE510 (lower panel) cells. Lamin A/C was served as internal control. (C) The lysates of indicated KYSE510 (left panel) or KYSE410 (right panel) cells were immunoprecipitated with Gli1 antibody (IP: Gli1), and then immunoblotted with antibodies against CCR4 (IB: CCR4), AKT (IB: AKT), FAK (IB: FAK), DGKα (IB: DGKα), and Gli1 (IB: Gli1). (D) The lysates of indicated KYSE510 (left panel) or KYSE410 (right panel) cells were immunoprecipitated with suppressor of fused homolog (SuFu) antibody (IP: SuFu), and then immunoblotted with antibodies against CCR4 (IB: CCR4), AKT (IB: AKT), FAK (IB: FAK), DGKα (IB: DGKα), and SuFu (IB: SuFu). (E) Immunoprecipitation‐immunoblotting (IP‐IB) assay for the interaction between Gli1 and AKT (IP: Gli1, and then IB: Gli1; IB: AKT) in indicated KYSE510 (upper panel) or KYSE410 (lower panel) cells. (F) IP‐IB assay for the interaction between Gli1 and HA‐AKT (IP: HA, and then IB: Gli1; IB: HA) in indicated KYSE410 (upper panel) or KYSE510 (lower panel) cells. (G) Gli transcriptional activity was evaluated using luciferase reporter assay. The treatment condition of (G) was similar to (F). Error bars, mean ± SD of three independent experiments.

HH inhibitors, such as GDC‐0449 and cyclopamine (2.5 μM), made no difference to rhCCL22 (50 ng/mL)‐induced Gli1 activation or nuclear accumulation in KYSE410 and KYSE510 cells (Figure 3A,B), indicating that CCL22 activates Gli1 independent of SMO. We further evaluated if FAK (downstream effector of CCL22/CCR4 axis) participated in regulating CCL22‐stimulated Gli1 activity using FAK inhibitor‐VS‐4718, and found that VS‐4718 (1 μM) blocked rhCCL22‐stimulated Gli1 nuclear location and activity (Figure 3A,B), indicating that CCL22 activates Gli1 might require the activation of FAK. Furthermore, VS‐4718 (1 μM), but not cyclopamine and GDC‐0449 (2.5 μM), blocked the upregulation of SOX2, Nanog, and OCT4 levels induced by rhCCL22 (50 ng/mL) in KYSE410 and KYSE510 cells (Figure S7A–C). FAK siRNA was transfected into ESCC cells, which were then incubated with rhCCL22 (50 ng/mL) to measure Gli1 function. As the results shown in Figure S8A,B, FAK siRNA effectively inhibited CCL22‐stimulated Gli1 activity and nuclear expression in indicated ESCC cells.

For analyzing the role of CCL22/FAK axis in activating Gli1 function, we assessed the interaction of Gli1 with the FAK‐related signalosome components.^14^ Without the stimulation of CCL22, no interactions were found between Gli1 and CCR4/DGKα/FAK signalosome, only slight interaction between Gli1 and AKT. However, following rhCCL22 (50 ng/mL) stimulation, Gli1 strongly interacted with AKT rather than other components in the CCR4/DGKα/FAK signalosome (Figure 3C). Because Gli1 activity is suppressed by suppressor of fused homolog (SuFu), we analyzed if CCR4/DGKα/FAK axis interacted with SuFu. As the results shown in Figure 3D, SuFu showed no interaction with any components of this signalosome, regardless of CCL22 treatment. VS‐4718 (1 μM), but not cyclopamine and GDC‐0449 (2.5 μM), effectively disrupted the interaction between AKT and Gli1 (Figure 3E). Correspondingly, CCR4 or FAK siRNAs significantly abolished the CCL22‐induced formation of Gli1/AKT complex in indicated ESCC cells (Figure S9A,B). Taken together, the CCL22/FAK axis might regulate Gli1 via FAK/AKT axis.

### 2.4 Gli1 is phosphorylated by FAK/AKT axis

We evaluated whether AKT might be activated to interact with Gli1. Wild‐type (wt) AKT, constitutively activated AKT (S473/T308D), or function‐loss AKT (S473/T308A) was transfected into the KYSE410 and KYSE510 cells (Figure S10A). AKT wt and AKT S473/T308D increased AKT activity (Figure S10B,C) and facilitated the interaction between AKT and Gli1 (Figure 3F). VS‐4718 (1 μM) inhibited the activation of AKT and the interaction between AKT and Gli1 in indicated ESCC cells harbored AKT wt, but not significantly suppressed those of AKT S473/T308D (Figure 3F and Figure S10B,C). Additionally, ectopic expression of AKT S473/T308A showed no interaction with Gli1 (Figure 3F). Importantly, AKT and AKT S473/T308D ectopic expression, but not AKT S473/T308A, enhanced Gli activity (Figure 3G). VS‐4718 (1 μM) effectively blocked the stimulatory effect of AKT, but not AKT S473/T308D on Gli activity (Figure 3G). Therefore, only activated AKT can form the complex with Gli1 while enhancing its activity.

### 2.5 Phosphorylated Gli1 inhibits SuFu binding

**FIGURE 4:**
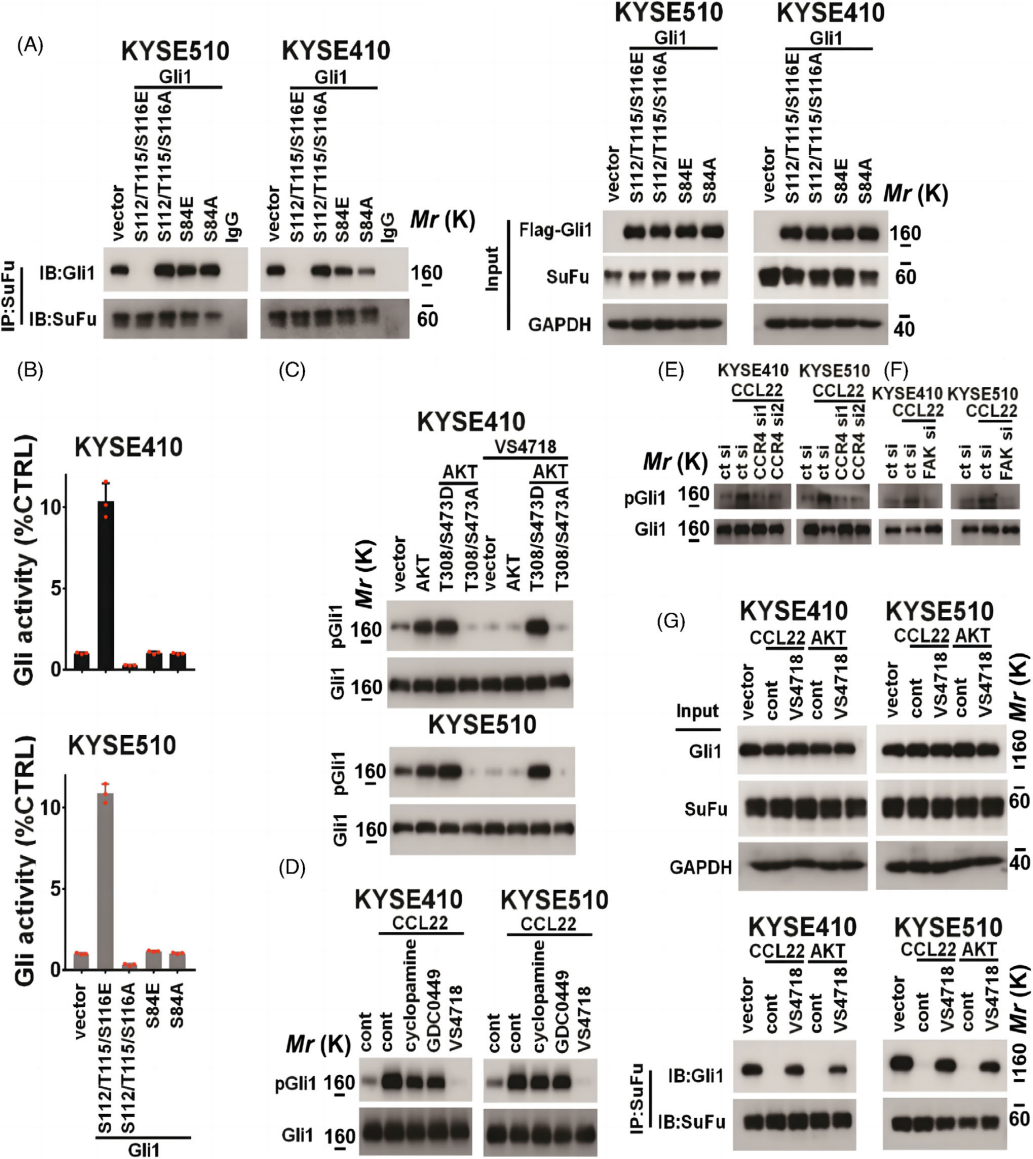
C‐C motif chemokine 22 (CCL22) **facilitates the phosphorylation of** glioma‐associated oncogene 1 (Gli1). (A) The interaction between Gli1 and SuFu was assessed by Immunoprecipitation‐immunoblotting (IP‐IB) analysis in indicated KYSE510 (left panel) and KYSE410 (right panel) cells harbored indicated Gli1 mutants. Input was shown. (B) The Gli transcriptional activity of KYSE410 (upper panel) and KYSE510 (lower panel) cells harbored indicated Gli1 mutants was evaluated using luciferase reporter assay. (C) The expression of pGli1 Ser^112^/Thr^115^/Ser^116^ and Gli1 in KYSE410 (upper panel) or KYSE510 (lower panel) cells harbored indicated AKT mutants with/without VS‐4718 (1 μM) was evaluated using immunoblotting. (D) The expression of pGli1 Ser^112^/Thr^115^/Ser^116^ and Gli1 in indicated KYSE410 (left panel) or KYSE510 (right panel) cells was evaluated using immunoblotting. (E–F) KYSE410 and KYSE510 cells harbored control siRNA or CCR4 siRNAs (E) or focal adhesion kinase (FAK) siRNA (F) were incubated with rhCCL22 (50 ng/mL), and the expression of pGli1 Ser^112^/Thr^115^/Ser^116^ and Gli1 was evaluated using immunoblotting. (G) The interaction between Gli1 and SuFu in indicated KYSE410 (left panel) and KYSE510 (right panel) cells was assessed by IP‐IB analysis (IP: SuFu; IB: SuFu, Gli1). Input was shown.

N‐terminal in Gli1 has a critical role in the interaction of Gli1 with SuFu, and phosphorylation of Gli1 N‐terminal induces the dissociation of Gli1 from Gli1/SuFu complex.^22^, ^30^, ^31^ Gli1 N‐terminal includes two potential AKT‐recognized motifs (RxxS/T), including 80‐KRALSIS‐86 or 111‐NSRCTSP‐117 (https://www.uniprot.org/uniprotkb/P08151/entry). We then investigated whether the phosphorylation of Gli1 Ser^84^ or Ser^112/^Thr^115/^Ser^116^ sites affects Gli1 binding with SuFu. Gli1 S112/T115/S116E, S112/T115/S116A, S84E, or S84A mutant was, respectively, transfected into KYSE410 and KYSE510 cells, and interaction of Gli1 with SuFu was evaluated by immunoprecipitation assay. In Figure 4A, Gli1 S112/T115/S116E mutant facilitated the dissociation of Gli1 from the SuFu/Gli1 complex, whereas mutation of S84 in Gli1 produced no effect on interaction between Gli1 with SuFu (Figure 4A). The above findings indicated that Ser^112/^Thr^115/^Ser^116^ phosphorylation within Gli1 might decrease SuFu binding. Correspondingly, Gli1 S112/T115/S116E facilitated the activity of Gli (Figure 4B) and upregulated the expression of SOX2, Nanog, and OCT4 in indicated ESCC cells (Figure S11A–C). Gli1 S112/T115/S116A inhibited Gli activity and the level of these stemness‐related markers; however, Gli1 S84E or S84A mutant produced no obvious effect on Gli activity and the expression of SOX2, Nanog, and OCT4 (Figure 4B and Figure S11A–C).

The phosphorylation of Gli1 Ser^112/^Thr^115/^Ser^116^ sites was evidently observed with the ectopic expression of AKT and AKT S473/T308D, but not the AKT S473/T308A (Figure 4C). VS‐4718 (1 μM) blocked the phosphorylation of Gli1 Ser^112/^Thr^115/^Ser^116^ sites in AKT wt, but not AKT S473/T308D ESCC cells (Figure 4C). Furthermore, inhibition of FAK, rather than SMO pathway, could block the phosphorylation of Gli1 Ser^112/^Thr^115/^Ser^116^ induced by CCL22 (Figure 4D). CCR4 or FAK siRNAs inhibited the CCL22‐induced phosphorylation of Gli1 Ser^112/^Thr^115/^Ser^116^ sites in indicated ESCC cells (Figure 4E,F). These trends were consistent with Gli1 activity (Figures S3 and S8). Additionally, either AKT ectopic expression or rhCCL22 (50 ng/mL) administration reduced SuFu binding to Gli1 (Figure 4G). This effect was totally reversed by VS‐4718 treatment (Figure 4G).

Furthermore, ESCC cells harbored Gli1 S112/T115/S116E mutant had the strongest invasive and anchorage‐independent growth abilities among all transfected cells (Figure S12A,B and Figure S13). Gli1 wt also facilitated the invasion and soft agar‐based colony formation in KYSE410/KYSE510 cells, especially under the stimulation of CCL22‐positive TAMs and rhCCL22 (Figure S12A,B and Figure S13). However, Gli1 S112/T115/S116A mutant decreased the invasion and anchorage‐independent growth of indicated ESCC cells, compared with control vector (Figure S12A,B and Figure S13).

Importantly, IHC analysis in 114 cases of clinical ESCC samples showed that pGli1 Ser^112^/Thr^115^/Ser^116^ was abundantly expressed in tumor cells (71%; 81 out of 114), but it showed negative expression within adjacent non‐carcinoma esophageal samples (24%; pGli1 positive samples were 16 out of 66) (Figure S14A). Strong expression of pGli1 Ser^112^/Thr^115^/Ser^116^ was positively related to several clinical parameters, including advanced stage, lymph node status, and high‐grade tumor status (Figure S14B). Results of Kaplan–Meier curves showed that the upregulated pGli1 Ser^112^/Thr^115^/Ser^116^ level exhibited negative relation to ESCC survival (Figure S14C). Specifically, analysis of 45 cases of clinical ESCC samples showed that stromal CCL22 exhibited positive relation to intratumoral expression of pGli1 Ser^112^/Thr^115^/Ser^116^ (Figure S14D).

### 2.6 The combination treatment targeting FAK and Gli1 within ESCC cells generates superior efficacy

**FIGURE 5:**
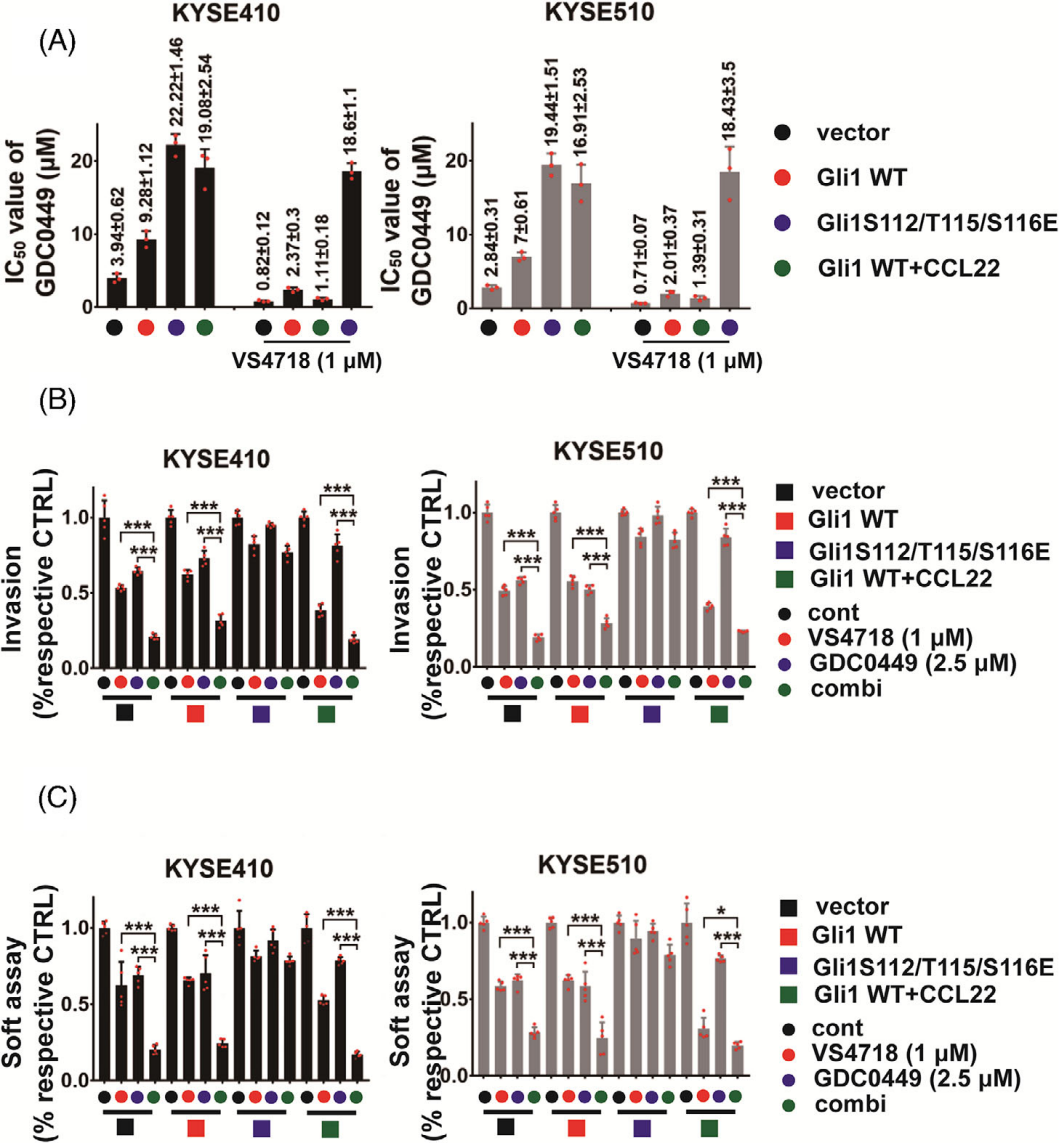
**Combination of VS‐4718 and GDC‐0449 in** esophageal squamous cell carcinoma (ESCC) **treatment in vitro**. (A) KYSE410 (left panel) or KYSE510 (right panel) cells harbored control vector, wild‐type glioma‐associated oncogene 1 (Gli1) with/without rhCCL22 (50 ng/mL), Gli1 S112/T115/S116E mutant, were treated with GDC‐0449 (0‐25 μM) alone or in the presence of VS‐4718 (1 μM) for 3 days. The proliferation was evaluated using MTS assay. The IC_50_ value of GDC‐0449 was listed. (B) Boyden chamber assay for KYSE410 (left panel) or KYSE510 (right panel) cells harbored control vector, wild‐type Gli1 with/without rhCCL22 (50 ng/mL; in the lower chamber), and Gli1 S112/T115/S116E mutant were plated on the upper cell culture inserts and treated with GDC‐0449 (2.5 μM), VS‐4718 (1 μM), and their combination. (C) Soft agar‐based colony formation for KYSE410 (left panel) or KYSE510 (right panel) cells harbored control vector, wild‐type Gli1 with or without rhCCL22 (50 ng/mL), and Gli1 S112/T115/S116E mutant were treated with GDC‐0449 (2.5 μM), VS‐4718 (1 μM), and their combination for 8 days. *p < 0.05; ***p < 0.001 as compared with respective control cells. Error bars, mean ± SD of three to five independent experiments.

Because Gli1 activation regulated by CCL22/FAK axis was independent of SMO, this axis‐activated Gli1 might promote HH inhibitor resistance in ESCC cells. Actually, we found that the HH inhibitor (GDC‐0449) IC_50_ level (0–25 μM) in ESCC/Gli1 S112/T115/S116E (GDC‐0449 IC_50_ level in KYSE410/Gli1 S112/T115/S116E cells was 22.22 ± 1.46 μM; in KYSE510/Gli1 S112/T115/S116E cells, GDC‐0449 IC_50_ level was 19.44 ± 1.51 μM) was much higher than that in ESCC/control vector (the IC_50_ value of GDC‐0449 in KYSE410 control vector cells was 3.94 ± 0.62 μM; in KYSE510 control vector cells, the IC_50_ value was 2.84 ± 0.31 μM) and ESCC/wt Gli1 (the IC_50_ value of GDC‐0449 in KYSE410/Gli1 wt cells was 9.28 ± 1.12 μM; in KYSE510/Gli1 wt cells, the IC_50_ value was 7 ± 0.61 μM) (Figure 5A). rhCCL22 (50 ng/mL) treatment increased GDC‐0449 IC_50_ level in ESCC/wt Gli1 cells treatment (GDC‐0449 IC_50_ level in KYSE410/Gli1 wt cells was 19.08 ± 2.54 μM; in KYSE510/Gli1 wt cells, GDC‐0449 IC_50_ level was 16.91 ± 2.53 μM) (Figure 5A).

We further evaluated whether a combination of inhibition of SMO and FAK would be more effective for ESCC treatment. VS‐4718 (1 μM) significantly promoted the ability of GDC‐0449 in suppressing growth of ESCC cells (GDC‐0449 IC_50_ level in KYSE410 cells was 0.82 ± 0.12 μM; in KYSE510 cells, GDC‐0449 IC_50_ level was 0.71 ± 0.07 μM) and in these two ESCC cell lines harbored Gli1 wt alone (the IC_50_ value of GDC‐0449 in KYSE410/Gli1 wt cells was 2.37 ± 0.3 μM; in KYSE510/Gli1 wt cells, the IC_50_ value was 2.01 ± 0.37 μM) or with 50 ng/mL rhCCL22 (GDC‐0449 IC_50_ value in KYSE410/Gli1 wt cells in the presence of CCL22 was 1.11 ± 0.18 μM; in KYSE510/Gli1 wt cells in the presence of CCL22, GDC‐0449 IC_50_ value was 1.39 ± 0.31 μM) (Figure 5A). Importantly, the sensitizing effect of VS‐4718 on GDC‐0449 was not observed in ESCC/Gli1 S112/T115/S116E cells (GDC‐0449 IC_50_ level in KYSE410/Gli1 S112/T115/S116E cells was 18.6 ± 1.1 μM; in KYSE510/Gli1 S112/T115/S116E cells, GDC‐0449 IC_50_ level was 18.43 ± 3.5 μM), indicating that FAK inhibition enhances the ability of HH inhibitor to suppress tumor development in vitro via suppressing the phosphorylation of Gli1 Ser^112/^Thr^115/^Ser^116^ sites (Figure 5A). Likewise, invasion and anchorage‐independent colony forming assays came to similar trends (Figure 5B,5C and Figure S15).

**FIGURE 6:**
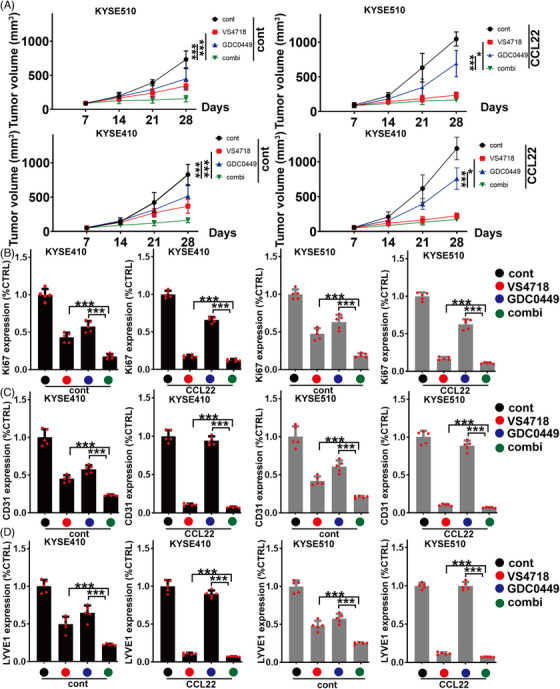
**Combination of VS‐4718 and GDC‐0449 in** esophageal squamous cell carcinoma (ESCC) **treatment in vivo**. (A) KYSE510 (upper panel) or KYSE410 (lower panel) tumor‐beared mice with (right panel)/without (left panel) rhCCL22 were treated with VS‐4718 or GDC‐0449 and their combination. Curves of tumor volumes were shown. (B–D) The expression of Ki‐67 (B), CD31 (C), or LYVE1 (D) in indicated KYSE410 and KYSE510 tumors was evaluated using quantitative enzyme‐linked immunosorbent assay (ELISA) assays. Black bar: KYSE410 tumor, left panel of KYSE410 group: cont (without C‐C motif chemokine 22 [CCL22]), right panel of KYSE410 group: with CCL22; gray bar: KYSE510 tumor, left panel of KYSE510 group: cont (without CCL22), right panel of KYSE510 group: with CCL22; *p < 0.05; ***p < 0.001 as compared with the control cells. Error bars, mean ± SD of five independent experiments.

We further examined whether inhibition of crosstalk between CCL22/FAK signalosome and HH pathway could block ESCC malignancy *in vivo. KY*SE410 and KYSE510 cells were injected into each mouse through subcutaneous inoculation, followed by GDC‐0449 (25 mg/kg/day, p.o.), VS‐4718 (20 mg/kg/day, p.o.), or their combination treatment. It was found that the combination of GDC‐0449 and VS‐4718 produced synergistic inhibitory effect on tumor growth than each agent alone (Figure 6A). Furthermore, co‐inhibition of FAK and Gli1 efficiently suppressed Ki67, CD31, and LYVE1 levels in ESCC tumors than each agent alone (Figure 6B–D). We observed whether co‐inhibition of FAK and Gli1 could inhibit CCL22‐mediated tumor malignancy. When xenografts reached approximate 100 mm^3^, animals were routinely treated with 0.1 μg/kg rhCCL22 (twice weekly, i.v.) with or without GDC‐0449, VS‐4718 alone, or their combination. Combination of GDC‐0449 and VS‐4718 could induce significantly synergistic inhibitory effect on CCL22‐stimulated tumor growth (Figure 6A) and those aforementioned tumor‐promoting biomarkers in indicated tumors (Figure 6B–D). Thus, the combination of these two inhibitors targeting FAK and HH pathways could produce an alternative targeted therapy for ESCC treatment in preclinical study.

## 3 DISCUSSION

We previously reported that TAMs‐released CCL22 facilitates ESCC malignancy via FAK‐based signalosome.^14^ The present work suggested that Gli1 was activated independent of SMO via FAK/AKT pathway, where the activated AKT phosphorylated Gli1 at Ser^112/^Thr^115/^Ser^116^ sites, causing the dissociation of Gli1 from SuFu binding and enhancement of its activity, and resultantly inducing stemness and metastasis of ESCC. These results support that TAMs‐derived CCL22 is the key chemokine for stimulating ESCC malignancy.

Identification of critical intratumoral signaling pathways regulated by TME has yielded potential strategies to target solid tumors.^32^, ^33^, ^34^, ^35^ TAMs, abundant components in the TME, release several cytokines and chemokines like IL‐6, CCL18, and IL‐8 for interacting with their respective receptors on cancer cells, thus activating oncogenic pathways and inducing tumor malignancy.^36^, ^37^, ^38^ According to our results, TAMs‐released CCL22 stimulates the CCR4/FAK/AKT/Gli1 signaling in ESCC cells, providing a novel TAMs/intratumoral axis in tumor progression. Our clinical ESCC samples analyses showed that intratumoral Gli1 can serve as biomarker for evaluating ESCC malignancy and ESCC prognosis, and positively correlates to the stromal level of CCL22.

The signaling pathway‐related molecular mechanism in tumor cells is extensively complex.^39^, ^40^, ^41^, ^42^ It remains challenging to understand crosstalk mechanisms among these signaling networks to develop targeted therapies. FAK functions as the convergence to coordinately link intracellular and intercellular signalings to facilitate tumor malignant progression.^15^, ^17^, ^18^, ^43^ In present study, our data identified that TAMs‐released CCL22 could act as a stress to stimulate Gli1 activity. Mechanistically, in response to CCL22 stimulation, FAK induced AKT to interact with Gli1 and then phosphorylate Gli1 on N‐terminal Ser^112^/Thr^115^/Ser^116^ sites, resulting in Gli1 release from its endogenous inhibitor‐SuFu binding and enhance its transcriptional activity independent of SMO. Our observations provide the novel phosphorylation sites in Gli1 for facilitating its transcriptional activity, and extend prior studies indicating the signaling proteins‐mediated Gli1 activation. Interestingly, our results showed that Gli1 did not interact with other components of CCL22/FAK signalosome, except for AKT, solidifying the result that Gli1 as a substrate for FAK/AKT pathway and the mechanisms for SMO‐independent Gli1 activation. Importantly, disruption of CCL22/FAK signalosome effectively blocked the phosphorylation and activation of Gli1. Mutation of Ser^112^/Thr^115^/Ser^116^ sites in Gli1 is sufficient to facilitate/abolish SuFu's inhibition against Gli1 and the signaling from upstream FAK.

FAK targeting is efficient when it is combined with additional agents for enhancing the efficacy of targeted therapy, chemotherapy, or immunotherapy in solid tumors.^18^, ^44^ For identifying cases who may gain the greatest beneficial effects from FAK inhibitor‐based combinatorial therapy, it is necessary to understand the interaction between FAK signaling and other druggable oncogenic proteins and relevant molecular mechanisms. HH pathway is recognized to be the therapeutic target in various tumors, such as ESCC. Some SMO inhibitors, especially GDC‐0449, have been analyzed in clinical studies.^45^, ^46^, ^47^, ^48^ In the present work, GDC‐0449 inhibit ESCC cell growth in vivo and in vitro, supporting that GDC‐0449 could also be used for ESCC treatment. Furthermore, Gli1 was activated via FAK/AKT pathway independent of SMO, but it was not suppressed via SMO inhibitors and was responsible to FAK inhibitors. Safety and effectiveness of FAK inhibitors had been studied in several clinical trials; however, FAK inhibitors usually exert restricted effect on cancers as the monotherapy, but they synergistically act with cytotoxic or other targeted agents. Importantly, one Phase II study was designed for analyzing combinatorial effect of HH inhibitor‐vismodegib with FAK inhibitor‐GSK2256098 on progressive meningioma cases (ClinicalTrials.gov Identifier: NCT02523014, first posted:2015), providing the possibility of combination of FAK and Gli1 inhibitors in clinical tumor treatment. In our study, cotreatment FAK inhibitor with SMO inhibitor, VS‐4718 with GDC‐0449, exhibited the superior inhibition against cancer development, invasion, and stemness activity than each agent alone. Although SMO inhibitors display promising tumor‐suppressive effect, resistance development may possibly be related to the overactivation of upstream signaling pathways. Our data of AKT phosphorylating Gli1 while enhancing its activity offers the molecular mechanism for CCL22/FAK axis‐regulated and AKT‐mediated Gli1 activation independent of SMO. Therefore, combined therapy of inhibitors that target both pathways is the efficient way for treating ESCC, or even other tumors. Additionally, through the IHC analysis of clinical ESCC samples, the expression of Gli1 and pGli1 Ser^112^/Thr^115^/Ser^116^ was positively correlated with stromal CCL22, indicating that the above ESCC cases possibly experience canonical HH pathway as well as CCL22‐regulated Gli1 activation independent of SMO. According to these clinical results, such cases cannot fully respond to GDC‐0449 monotherapy; however, they can gain beneficial effects from combined therapy of inhibitors that target FAK and HH pathways. Thus, exploring a vast array of possible therapeutic combinations based on signaling crosstalk assists in the simultaneous targeting of the above pathways.

**FIGURE 7:**
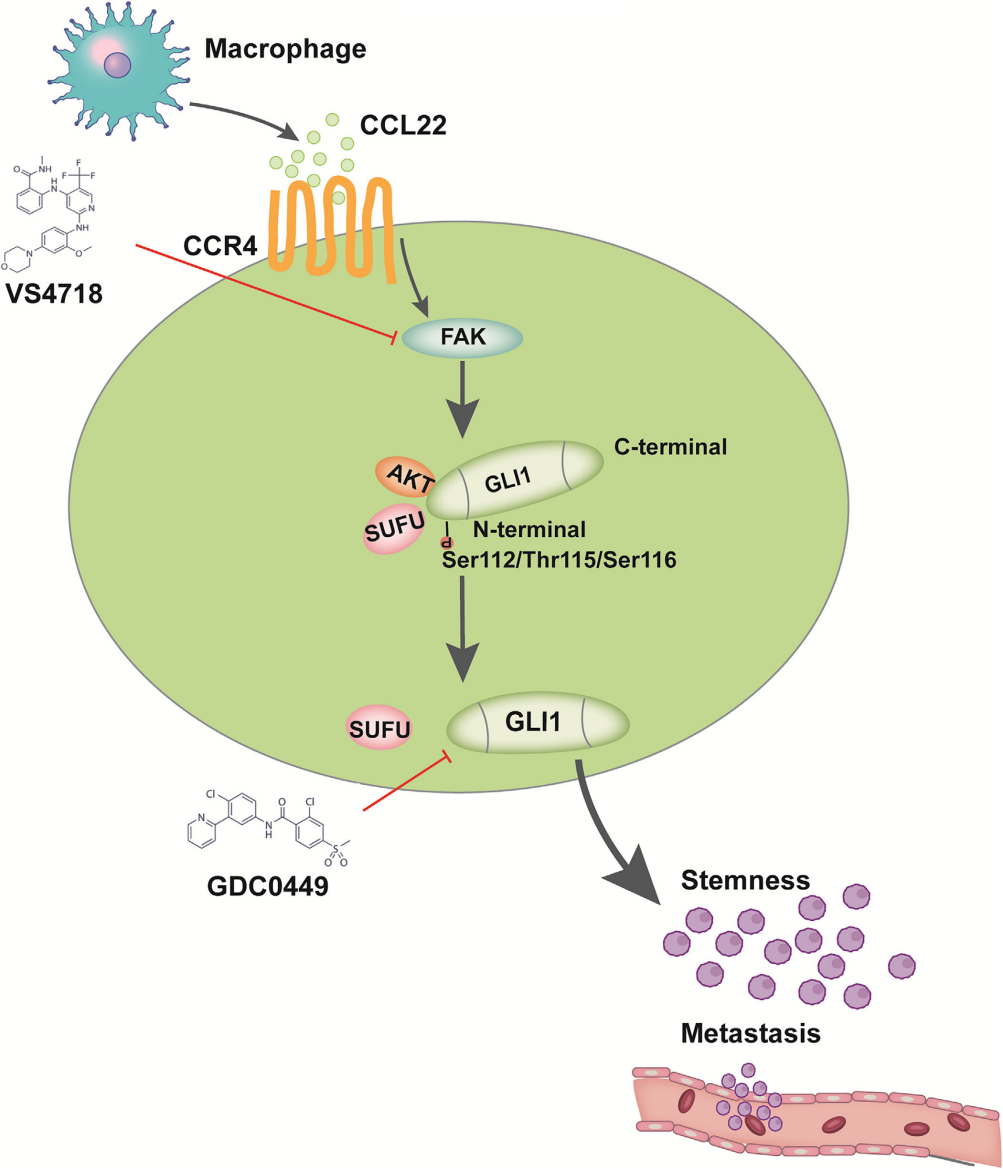
**Proposed model of** C‐C motif chemokine 22 (CCL22)**‐mediated the crosstalk between** focal adhesion kinase (FAK) **and** glioma‐associated oncogene 1 (Gli1) **in tumor cells**. Tumor‐associated macrophages (TAMs)‐released CCL22 activates intratumoral CCR4/FAK/AKT axis, which induces the phosphorylation of Gli1 Ser^112/^Thr^115/^Ser^116^ sites and facilitates Gli1 releases from suppressor of fused homolog (SuFu) to enhance Gli1 activity, and then induce the stemness and metastasis of tumor cells. FAK inhibitor‐VS‐4718 enhanced the antitumor effect of Hedgehog (HH) inhibitor‐GDC‐0449 both in in vitro and in vivo.

In summary, the present work identified Gli1 to be the substrate for CCL22/FAK/AKT axis, which provided the SMO‐independent Gli1 activation mechanism that was stimulated by TAMs‐derived chemokine. Our results also indicate that the combination of the inhibitors toward FAK and HH pathways potently suppressed ESCC with or without TAMs compared with monotherapy. Importantly, stromal CCL22 was related to intratumoral Gli1 in clinical ESCC samples, suggesting that the combined treatment that targeted FAK and HH pathways is efficient in treating ESCC, especially in the context of TME (Figure 7).

## 4 MATERIALS AND METHODS

### 4.1 Reagents and antibodies

Anti‐DGKα (Cat# H00001606‐B01P), anti‐CD204 (Cat# PAB30252), and anti‐Gli1 (Cat# MAB16740) antibodies were provided by Abnova. Anti‐FAK (Cat# 39−6500) antibody was obtained from Invitrogen. Antibodies against CCL22 (Cat# ab217351) and CCR4 (Cat# ab83250) were obtained from Abcam. Antibodies against AKT (Cat# 9272) and SuFu (Cat# 2522) were obtained from CST. Antibody against Lamin A/C (Cat# A17319) was obtained from ABclonal. pGli1 Ser^112^/Thr^115^/Ser^116^ antibody was synthesized from ABclonal.

### 4.2 Cell culture and transfection

Human primary TAMs extracted from ESCC tissues were according to prior work.^14^ Human KYSE410 and KYSE510 ESCC cells (provided by Dr. Shemada from Kyoto University) were first cultured with RPMI‐1640 medium that contained 1% penicillin‐streptomycin as well as 10% fetal bovine serum (FBS). The Gli1 shRNA1 and shRNA2, and indicated AKT or Gli1 mutants were stably transfected into KYSE410 or KYSE510 cells, and positive cells were selected for further studies. The sequences of CCR4 siRNAs and FAK siRNA were in line with our prior works.^14^, ^49^ The sequences of Gli1 shRNA1 and 2 were shown as follows:

Gli1 shRNA1: 5′‐GCGAGAAGCCACACAAGTGCACGTTTGAA‐3′;
Gli1 shRNA2: 5′‐TCTCACCTCTGTCGGATGCCAGCCTGGAC‐3′.

### 4.3 Cell viability

The 3‐(4,5‐dimethylthiazol‐2‐yl)‐5‐(3‐carboxymethoxyphenyl)‐2‐(4‐sulfophenyl)‐2H‐tetrazolium, inner salt (MTS) assay (Promega) was conducted to evaluate ESCC cell viability. Briefly, KYSE410 or KYSE510 cells were inoculated into 96‐well plates, followed by culture with/without specific agents for a 72‐h period prior to examination. After aspiration of medium, MTS solution was supplemented into cell cultures, followed by 1‐h incubation under 37°C. A microplate reader (TECAN) was utilized to measure absorbance (optical density; OD value) at 490 nm.

### 4.4 Soft agar colony formation assay

The cell transformation assay kit (Cat# K921‐100; Biovision) was utilized to analyze the anchorage‐independent growth of ESCC cells. Briefly, the KYSE410/KYSE510 cell suspensions mixed by 1.2% agarose solution and 1 × DMEM solution were introduced into 96‐well plate precoated with solidified agarose layer. Then, indicated agents or/and CCL22 (50 ng/mL) were treated for 8 days. Every well was introduced with DMEM solution containing WST working solution, followed by 4‐h incubation under 37°C. The microplate reader (TECAN) was employed to measure OD values of all wells at 450 nm and quantitatively analyze the anchorage‐independent growth ability of indicated ESCC cells. The exact protocol was followed as mentioned in our prior works.^50^, ^51^, ^52^

### 4.5 Invasion assay

ESCC cell invasive ability was evaluated by cell invasion assay kit (Cat # K912‐100; Biovision). Briefly, KYSE410/KYSE510 cells were added into top transwell chamber, and the indicated agents were also added to the top well. TAMs or CCL22 (50 ng/mL) was introduced into bottom transwell chamber. After 24 h, uninvaded cells were removed out of top chamber with the cotton swab, invaded cells were obtained using cell dissociation solution, and incubated with invasion assay kit‐contained cell dye at 37°C for 60 min. The invasive cells were then quantified using a fluorometer. The exact protocol was followed as mentioned in our prior works.^14^, ^51^, ^52^ The crystal violet staining of invaded ESCC cells was also applied according to our previous studies.^14^, ^50^

### 4.6 Luciferase reporter assay

The Gli luciferase reporter assay is applied to evaluate the transcriptional activity of Gli. Briefly, after inoculation of KYSE410 and KYSE510 cells onto the 24‐well plate, the pGLI luciferase reporter plasmid (containing multi‐sites of GLI‐responsive element) was transfected into indicated KYSE410 and KYSE510 cells. Subsequently, the indicated ESCC cells harbored pGLI luciferase reporter vector has been lysed by luciferase assay‐related cell lysis solution and then subjected to firefly luciferase reporter assay to assess luciferase activity (measurement of the amount of conversion from luciferin to oxyluciferin).

### 4.7 Quantitative ELISA assays

Human CCL22/MDC ELISA kit (R&D Systems; Cat# DMD00) was applied to assess the secretion of CCL22 from TAMs (six cases). To evaluate AKT activity in indicated ESCC cells, PathScan phospho‐AKT (Thr^308^; Cat# 7252; CST) and phospho‐AKT (Ser^473^; Cat# 7160; CST) sandwich ELISA kits were used to evaluate the regulatory effect of several AKT mutants with/without VS‐4718 (1 μM) on the intratumoral concentration of pAKT Ser^473^ or Thr^308^. Human Sox2 ELISA kit (CST; Cat# 7277), human Nanog ELISA kit (Abcam; Cat# ab236720), and human OCT‐4 ELISA kit (Raybiotech; Cat# ELH‐OCT4‐1) were applied to measure expression of stemness‐related biomarkers, including SOX2, Nanog, and OCT4 in indicated ESCC cells. The exact experimental procedures of above ELISA kits were in line with respective manufacturer's instruction.

### 4.8 Immunoprecipitation and immunoblotting

Immunoprecipitation‐immunoblotting (IP‐IB) assays were applied to assess the generation of Gli1‐related protein complex. After harvesting, cells were rinsed by phosphate‐buffered solution (PBS), lysed with IP lysis buffer containing protease/phosphatase inhibitors for 40 min. Cell extracts were harvested prior to 10‐min centrifugation at 10,000 *g* and 4°C. Then, lysates were incubated with indicated antibodies as well as protein A/G agarose (Thermo; Cat#20421) onto the rotator overnight under 4°C. Immunocomplexes were washed five times using lysis buffer, followed by 5‐min boiling within SDS‐loading buffer. Finally, protein lysates were subjected to immunoblotting using specific antibodies. Immunoblotting assay protocols were the same as in our prior works.^14^, ^53^

### 4.9 Xenograft nude mice model

All mice were kept under the pathogen‐free condition under protocols that gained approval from the Animal Center, Peking University Cancer Hospital & Institute. Xenograft studies were conducted using female BALB/c nude mice (5‐weel‐old, Vital River Laboratories) and given subcutaneous injection of indicated ESCC cells in right flank. After tumor reaching approximately 100 mm^3^ (*n* = 5/group), 0.1 μg/kg CCL22 (twice per week, i.v.) with/without VS‐4718 (20 mg/kg/day, p.o.) or/and GDC‐0449 treatment (25 mg/kg/day, p.o.) was given. Treatment was sustained 3 weeks. Tumor volumes were monitored by the following formula: mm^3^ = (Length × width^2^) × 0.5.

Human Ki‐67 (Raybiotech, Cat# ELH‐MKI67‐1), PECAM‐1 (CD31) (Raybiotech, Cat# ELH‐PECAM1‐1), and LYVE‐1 (Raybiotech, Cat# ELH‐LYVE1‐1) ELISA kits were applied to measure Ki‐67, CD31, and LYVE1 levels in indicated ESCC tumors. The exact experimental protocols of above indicated ELISA kits were in line with specific protocols.^14^, ^51^, ^52^

### 4.10 Clinical samples and IHC staining

Present work gained approval from institutional Review Board of Peking University Cancer Hospital (2018KT107). The IHC staining of ESCC samples was in line with our prior works, and other experimental protocols and the evaluation of staining intensity were described previously.^14^, ^51^, ^52^, ^53^ Primary antibody concentrations used in present study were as follows: CD204 (1:500), CCL22 (1:100), Gli1 (1:500), and pGli1 Ser^112^/Thr^115^/Ser^116^ (1:500).

### 4.11 Statistical analysis

GraphPad prism (GraphPad Software Inc.) was employed for statistical analysis. Experimental results were represented by mean ± standard deviation (SD) for statistical significance by unpaired Student's *t*‐test between two groups. *p* Value < 0.05 presents statistical significance.

## AUTHOR CONTRIBUTIONS

Qimin Zhan designed the experiments and wrote the paper. Jie Chen, Yanmeng Zhu, Di Zhao, Lingyuan Zhang, Jing Zhang, Yuanfan Xiao, Qingnan Wu, and Yan Wang performed the experiments and analyzed the data. All authors have read and approved the final manuscript.

## CONFLICT OF INTEREST STATEMENT

The authors declare no conflicts of interest.

## ETHICS STATEMENT

In this study, collection of clinical samples obtained approval from institutional Review Board of Peking University Cancer Hospital (2018KT107).

## Supporting information

Supporting Information

## Data Availability

The datasets used and/or analyzed during the current study are available from the corresponding author upon reasonable request.
