# Supplementary material for: Co‐targeting FAK and Gli1 inhibits the tumor‐associated macrophages‐released CCL22‐mediated esophageal squamous cell carcinoma malignancy
Source: MedComm (2020). 2023 Oct 15;4(6):e381. doi: 10.1002/mco2.381 (PMC10576977; doi:10.1002/mco2.381)
Supplement: Supplementary file 1 — Supporting Information [file MCO2-4-e381-s001.docx]

**Co-targeting FAK and Gli1 inhibits the tumor-associated macrophages-released CCL22-mediated esophageal squamous cell carcinoma malignancy**

Jie Chen^1,2,3,4#^, Yanmeng Zhu^1#^, Di Zhao^1,2,3^, Lingyuan Zhang^1^, Jing Zhang^1^, Yuanfan Xiao^1^, Qingnan Wu^1,2,3^, Yan Wang^1,2,3^ and Qimin Zhan^1,2,3,4,5^*

**Affiliations:**

^1^Key Laboratory of Carcinogenesis and Translational Research (Ministry of Education/Beijing), Laboratory of Molecular Oncology, Peking University Cancer Hospital & Institute, Beijing 100142, China.

^2^Peking University International Cancer Institute, Peking University, Beijing 100191, China

^3^Research Unit of Molecular Cancer Research, Chinese Academy of Medical Sciences, Beijing, China

^4^Soochow University Cancer Institute, Suzhou 215000, China

^5^Institute of Cancer Research, Shenzhen Bay Laboratory, Shenzhen 518107, China

^#^These authors made equal contributions to this work

*Corresponding authors: Qimin Zhan, E-mail: [zhanqimin@bjmu.edu.cn](mailto:zhanqimin@bjmu.edu.cn)

**Supplementary Figures and Figure legends**


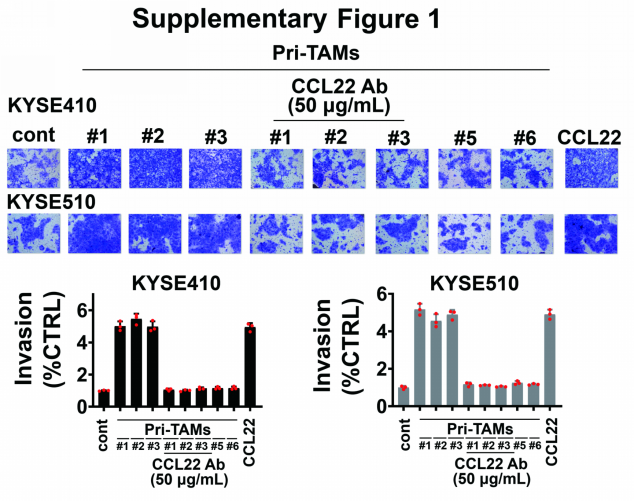


**Supplementary Figure 1. CCL22-positive TAMs facilitate the invasion of ESCC cells**

Transwell assay for KYSE410 (upper panel, representative images) or KYSE510 (lower panel, representative images) cells plated on the upper cell culture inserts, with culture CCL22-positive PriTAMs with/without CCL22 Ab, or CCL22-negative PriTAMs or rhCCL22 (50 ng/mL) in the lower chambers. After the experiment, crystal violet staining method was applied to evaluate the number of invaded KYSE410 and KYSE510 cells (statistical column charts were shown). Error bars, mean ± SD of three independent experiments.


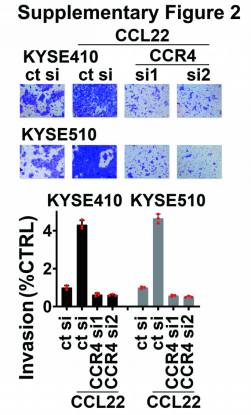


**Supplementary Figure 2. Intratumoral CCR4 is critical for CCL22-mediated ESCC invasion**

Transwell assay for KYSE410 (upper panel, representative images) and KYSE510 (lower panel, representative images) cells in the presence or absence of CCR4 siRNAs were treated with rhCCL22 (50 ng/mL). The invaded ESCC cells were observed by crystal violet staining method following transwell assay (statistical column charts were shown). Error bars, mean ± SD of three independent experiments.


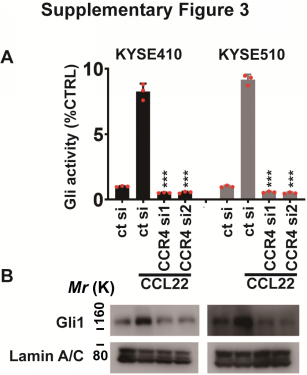


**Supplementary Figure 3. CCR4 is critical for CCL22-mediated Gli1 activation**

(A-B) The transcriptional activity and nuclear level of Gli1 of KYSE410 and KYSE510 cells harbored control siRNA or CCR4 siRNAs with rhCCL22 (50 ng/mL) were assessed by luciferase reporter assay (A) and immunoblotting assay (B). *** *P* < 0.001 as compared with the control cells. Two-tailed unpaired Student's *t*-test. Error bars, mean ± SD of three independent experiments.


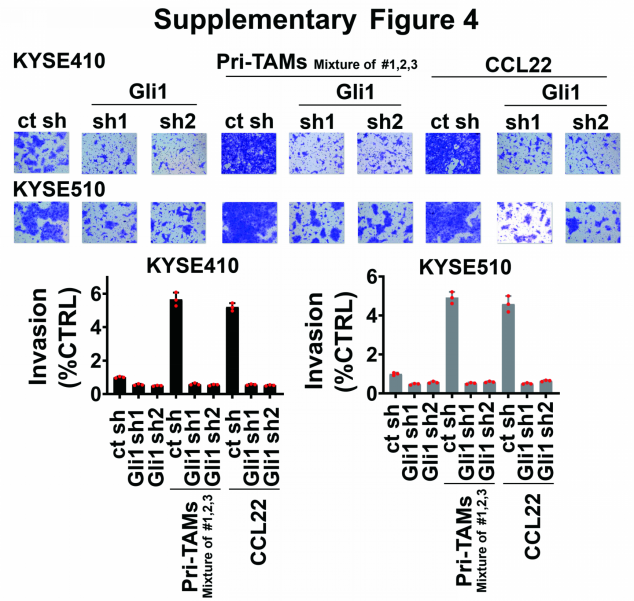


**Supplementary Figure 4. Gli1 mediates TAMs-released CCL22 -induced the invasion of ESCC cells**

Transwell assay for KYSE410 (upper panel, representative images) or KYSE510 (lower panel, representative images) cells plated on the upper cell culture inserts, with culture CCL22-positive PriTAMs or rhCCL22 (50 ng/mL) in the lower chambers. After the experiment, crystal violet staining method was applied to evaluate the number of invaded KYSE410 and KYSE510 cells (statistical column charts were shown). Error bars, mean ± SD of three independent experiments.


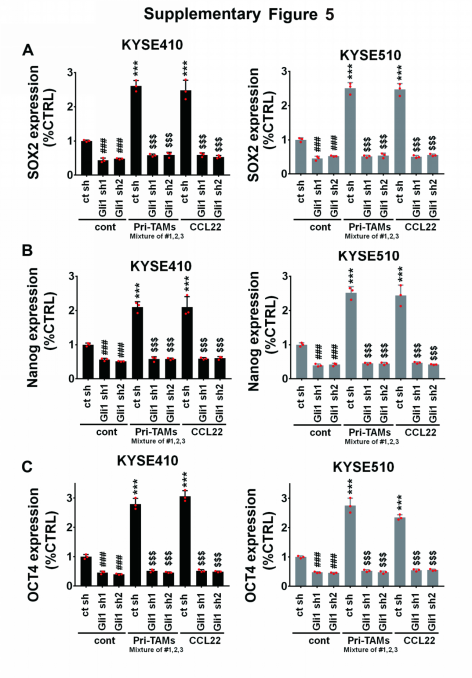


**Supplementary Figure 5. CCL22 facilitates the expression of stemness-related markers via Gli1**

(A-C) The expression of SOX2 (A), Nanog (B), and OCT4 (C) in indicated ESCC cells was evaluated using quantitative ELISA assays. *** *P* < 0.001 as compared with the control cells. $$$ *P* < 0.001 as respectively compared with PriTAMs CM group or rhCCL22 (50 ng/mL) group. ### *P* < 0.001 as compared with control cells. Error bars, mean ± SD of three independent experiments.


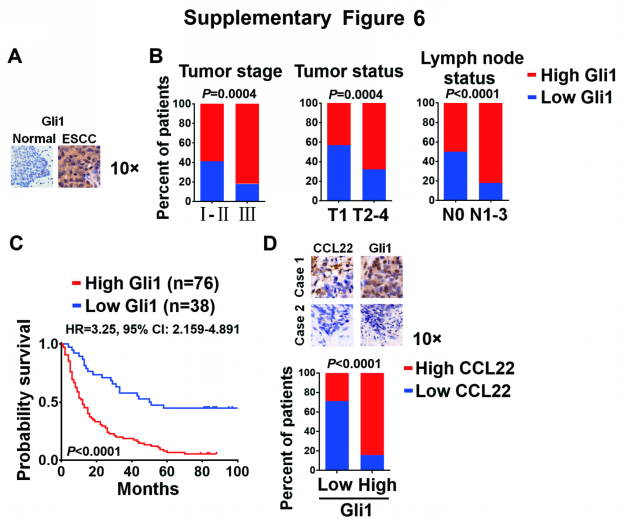


**Supplementary Figure 6. Intratumoral Gli1 clinically correlates with ESCC malignancy**

1. Immunohistochemical staining of ESCC samples indicating that Gli1 expression in human ESCC tissues compared with normal esophageal tissues. Representative images of IHC staining were shown. Magnification, 10 × as indicated. (B) Percentages of ESCC patients with high or low expression of Gli1 according to several clinical parameters, such as tumor stage (left), tumor status (middle), or lymph node status (right) (n=114). Two-tailed Pearson χ^2^ test. (C) Kaplan-Meier curves of ESCC patients with low vs high expression of Gli1 (n=114; *P* < 0.0001, log-rank test). (D) CCL22 expression was associated with intratumoral Gli1 expression in 45 primary human ESCC specimens. Two representative specimens with low and high levels of CCL22 expression were shown. Magnification, 10 × as indicated. Percentages of specimens showing low or high CCL22 expression relative to the level of intratumoral Gli1. Two-tailed Pearson χ^2^ test.


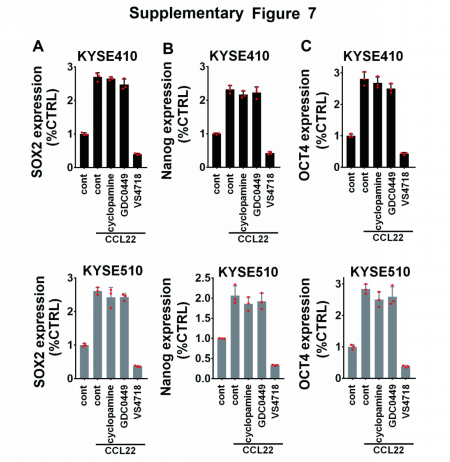


**Supplementary Figure 7. CCL22/FAK axis activates the expression of stemness-related markers**

(A-C) The expression of SOX2 (A), Nanog (B), and OCT4 (C) in indicated ESCC cells was evaluated using quantitative ELISA assays. Error bars, mean ± SD of three independent experiments.


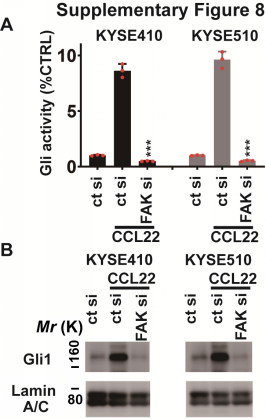


**Supplementary Figure 8. Intratumoral FAK is critical for CCL22-activated Gli1**

(A-B) The transcriptional activity and nuclear level of Gli1 of indicated ESCC cells harbored control or FAK siRNA with rhCCL22 (50 ng/mL) were assessed by luciferase reporter assay (A) and immunoblotting assay (B). *** *P* < 0.001 as compared with the control siRNA cells treated with rhCCL22 (50 ng/mL). Error bars, mean ± SD of three independent experiments.


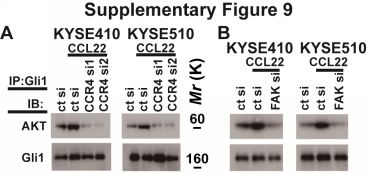


**Supplementary Figure 9. CCR4/FAK axis is contributed to CCL22-mediated the interaction between Gli1 and AKT**

(A-B) KYSE410 and KYSE510 cells harbored control siRNA or CCR4 siRNAs (A) or FAK siRNA (B) were incubated with rhCCL22 (50 ng/mL), and then subjected to IP-IB assay (IP: Gli1, and then IB: Gli1; IB: AKT) to evaluate the interaction between Gli1 and AKT.


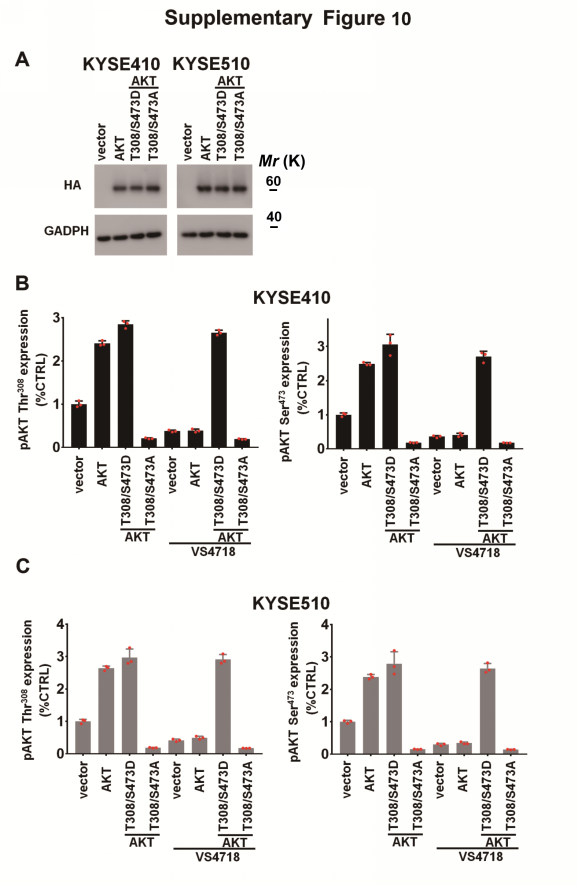


**Supplementary Figure 10. AKT activity in ESCC cells harbored several AKT mutant**

1. KYSE410 (left panel) and KYSE510 (right panel) cells were stably transfected with control vector, wild-type AKT, AKT S473/T308D, or AKT S473/T308A. The transfection efficacy was evaluated using immunoblotting assay. (B-C) KYSE410 (B) and KYSE510 (C) cells harbored control vector, wild-type AKT, AKT S473/T308D, or AKT S473/T308A were treated with/without VS-4718 (1 μM). The activity of AKT was quantified using phospho-AKT Thr^308^ and Ser^473^ ELISA assays. Error bars, mean ± SD of three independent experiments.


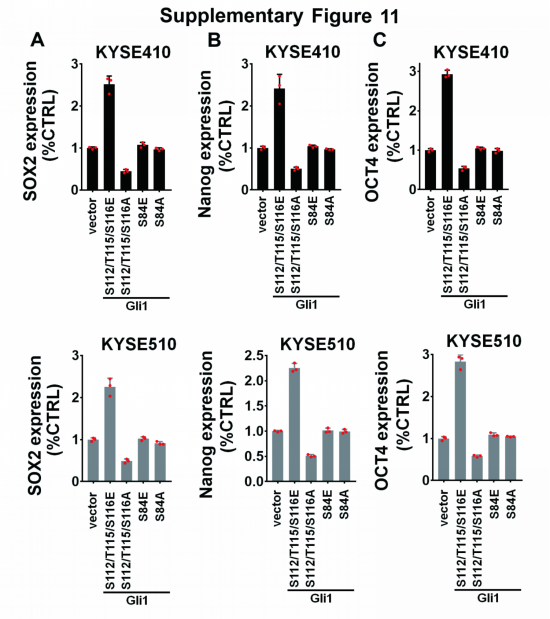


**Supplementary Figure 11. Activated Gli1 stimulates the expression of stemness-related markers**

(A-C) KYSE410 (upper panel) and KYSE510 (lower panel) cells harbored control vector, Gli1 S112/T115/S116E, or Gli1 S112/T115/S116A, Gli1 S84E or S84A mutant. The expression of SOX2 (A), Nanog (B), and OCT4 (C) in indicated ESCC cells was evaluated using quantitative ELISA assays. Error bars, mean ± SD of three independent experiments.


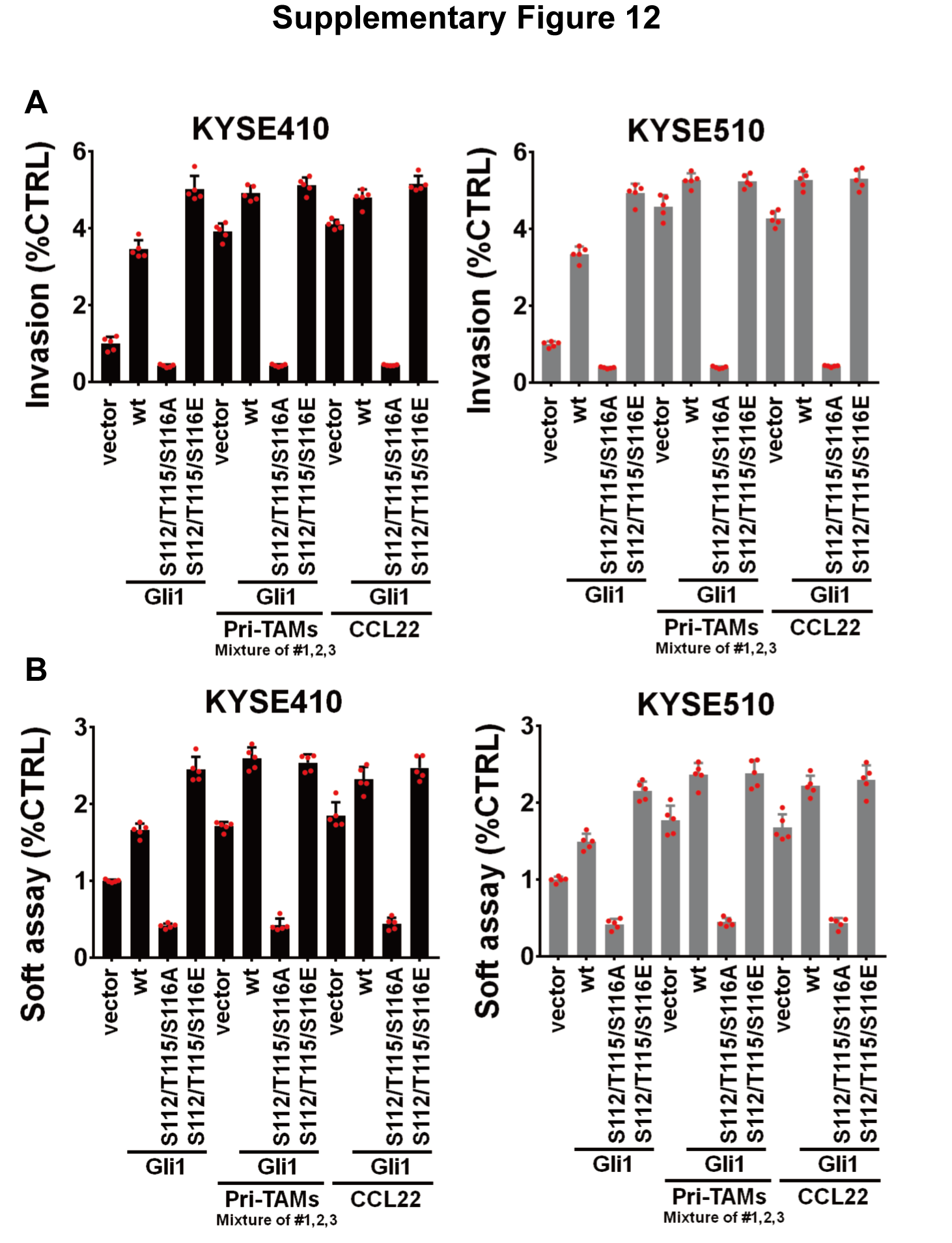


**Supplementary Figure 12.** **Phosphorylation of Gli1 Ser^112^/Thr^115^/Ser^116^** **sites** **is critical for ESCC malignancy**

1. Boyden chamber assay for KYSE410 (left panel) and KYSE510 (right panel) cells harbored control vector, wild-type Gli1, Gli1 S112/T115/S116E, or Gli1 S112/T115/S116A were plated on the upper cell culture inserts, with the PriTAMs (mixture of #1, 2, 3) or rhCCL22 (50 ng/mL) in the lower chambers. (B) Soft agar-based colony formation for indicated KYSE410 (left panel) or KYSE510 (right panel) cells treated with CM from PriTAMs (mixture of #1, 2, 3) or rhCCL22 (50 ng/mL) for 8 days. Error bars, mean ± SD of five independent experiments.


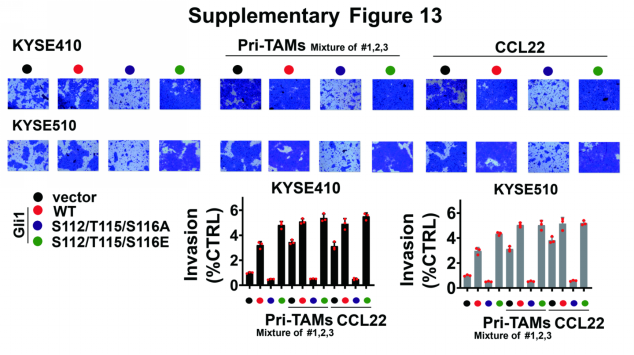


**Supplementary Figure 13. Phosphorylation of Gli1 Ser^112^/Thr^115^/Ser^116^** **sites** **is critical for ESCC invasion**

Transwell assay for KYSE410 (upper panel, representative images) and KYSE510 (lower panel, representative images) cells harbored control vector, wild-type Gli1, Gli1 S112/T115/S116E, or Gli1 S112/T115/S116A were plated on the upper cell culture inserts, with the PriTAMs (mixture of #1, 2, 3) or rhCCL22 (50 ng/mL) in the lower chambers. After the experiment, crystal violet staining method was applied to evaluate the number of invaded KYSE410 and KYSE510 cells (statistical column charts were shown). Error bars, mean ± SD of three independent experiments.


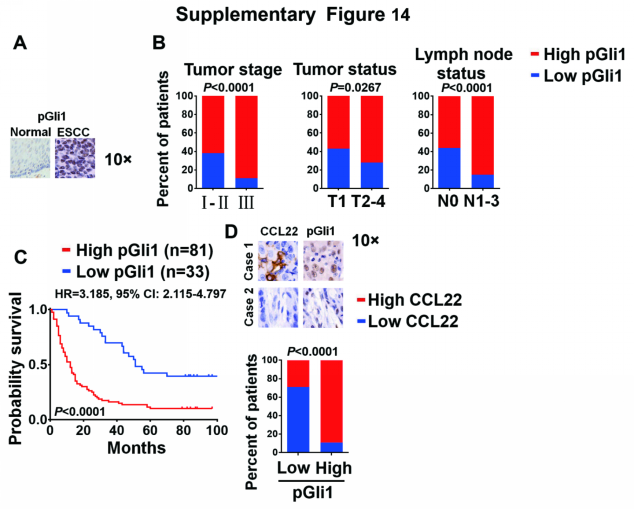


**Supplementary Figure 14. Intratumoral pGli1 clinically correlates with ESCC malignancy**

1. Immunohistochemical staining of ESCC samples indicating that pGli1 Ser^112^/Thr^115^/Ser^116^ expression in human ESCC tissues compared with normal esophageal tissues. Representative images of IHC staining were shown. Magnification, 10 × as indicated. (B) Percentages of ESCC patients with high or low expression of pGli1 Ser^112^/Thr^115^/Ser^116^ according to several clinical parameters, such as tumor stage (left), tumor status (middle), or lymph node status (right) (n=114). Two-tailed Pearson χ^2^ test. (C) Kaplan-Meier curves of ESCC patients with low vs high expression of pGli1 Ser^112^/Thr^115^/Ser^116^ (n=114; *P* < 0.0001, log-rank test). (D) CCL22 expression was associated with intratumoral pGli1 Ser^112^/Thr^115^/Ser^116^ expression in 45 primary human ESCC specimens. Two representative specimens with low and high levels of CCL22 expression were shown. Magnification, 10 × as indicated. Percentages of specimens showing low or high CCL22 expression relative to the level of intratumoral pGli1 Ser^112^/Thr^115^/Ser^116^. Two-tailed Pearson χ^2^ test.


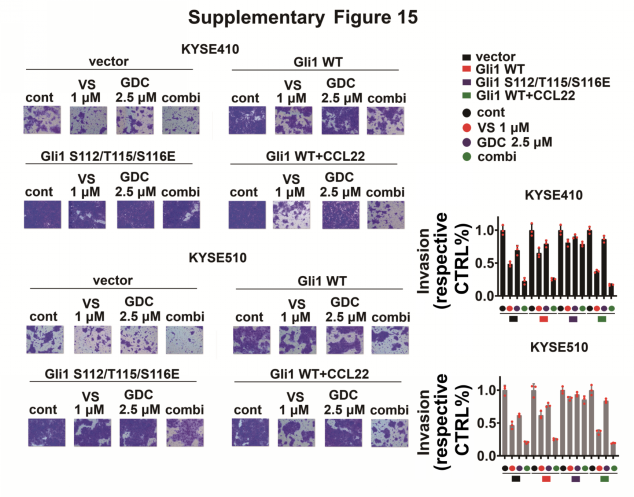


**Supplementary Figure 15. Phosphorylation of Gli1 Ser^112^/Thr^115^/Ser^116^** **sites is critical for the combinatorial effect of VS-4718 and GDC-0449 on ESCC invasion *in vitro***

Transwell assay for KYSE410 (upper panel, representative images) or KYSE510 (right panel, representative images) cells harbored control vector, wild-type Gli1 with/without rhCCL22 (50 ng/mL; in the lower chamber), Gli1 S112/T115/S116E mutant, were plated on the upper cell culture inserts and treated with GDC-0449 (2.5 μM), VS-4718 (1 μM), and their combination. After the experiment, crystal violet staining method was applied to evaluate the number of invaded KYSE410 and KYSE510 cells (statistical column charts were shown). Error bars, mean ± SD of three independent experiments.
